# Hydrophilic Pt nanoflowers: synthesis, crystallographic analysis and catalytic performance†
†Electronic supplementary information (ESI) available: TEM and high resolution HAADF-STEM analysis, FTIR and XRD measurements, and a summary of the catalytic performance for the different nanoparticles. See DOI: 10.1039/c6ce00039h



**DOI:** 10.1039/c6ce00039h

**Published:** 2016-04-12

**Authors:** Stefanos Mourdikoudis, Thomas Altantzis, Luis M. Liz-Marzán, Sara Bals, Isabel Pastoriza-Santos, Jorge Pérez-Juste

**Affiliations:** a Departamento de Química Física , Universidade de Vigo , 36310 Vigo , Spain; b EMAT , University of Antwerp , Groenenborgerlaan 171 , B-2020 Antwerp , Belgium . Email: pastoriza@uvigo.es ; Email: juste@uvigo.es; c BioNanoPlasmonics Laboratory , CIC biomaGUNE , Paseo de Miramón 182, 20009 San Sebastián , Spain; d Ikerbasque , Basque Foundation for Science , 48013 Bilbao , Spain

## Abstract

Water-soluble Pt nanoflowers (NFs) were prepared by diethylene glycol-mediated reduction of Pt acetylacetonate (Pt(acac)_2_) in the presence of polyethylenimine. Advanced electron microscopy analysis showed that the NFs consist of multiple branches with a truncated cubic morphology and different crystallographic orientations. We demonstrate that the nature of the solvent strongly influences the resulting morphology. The catalytic performance of the Pt NFs in 4-nitrophenol reduction was found to be superior to that of other nanoparticle-based catalysts. Additionally, the Pt NFs display good catalytic reusability with no loss of activity after five consecutive cycles.

## Abstract 2

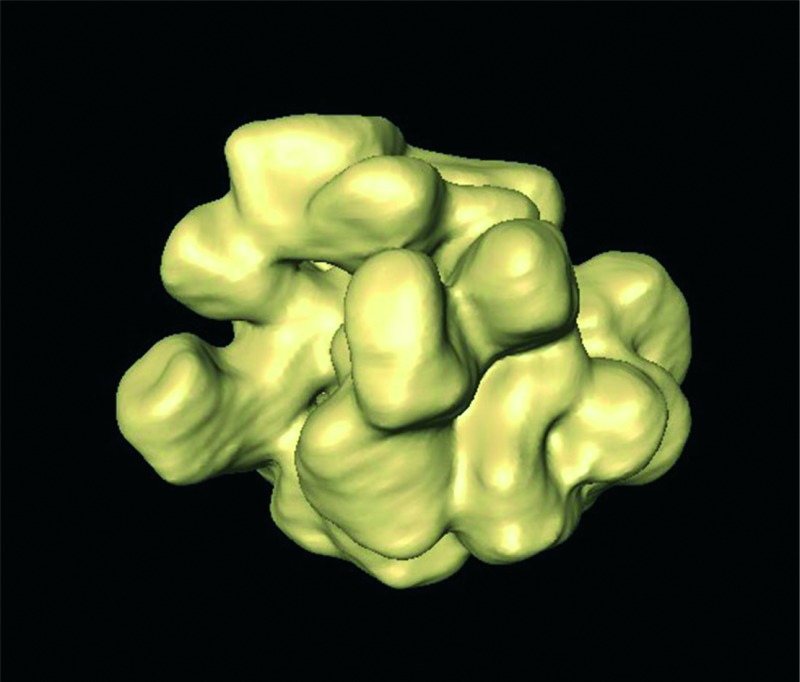
A 3D-tomography reconstructed image of a water-soluble Pt nanoflower, synthesized in diethyleneglycol in the presence of PEI.

## Introduction

Platinum nanostructures are important in domains such as catalysis, sensors and fuel cells.^1–5^ In this context, a significant amount of work has been devoted to tailoring their morphology by properly tuning the experimental reaction conditions.^6–8^ In this manner, shapes such as cubes, rods, dendrites, wires and polyhedra have been reported. In particular, the nanoflower configuration has attracted attention due to its surface roughness, excellent electrochemical performance and potential catalytic activity. Several synthetic routes toward Pt NFs have been reported in the literature, which usually yield hydrophobic Pt NFs.^7,9^ Sun *et al.* ^10^ reported a facile and large-scale synthesis of 100–200 nm 3D flower-like particles in aqueous medium, without using any capping agent, thus rendering them sensitive to aggregation. Other reports described the chemical synthesis of flower-like Pt nanoparticles in water, which were directly deposited on carbon nanotubes^11^ and the urea-assisted preparation of flower-like Pt arrays on other substrates.^6^ Monomorphic single-crystalline platinum nanoflowers, soluble in ethanol, were obtained by Zhang and co-workers through an iodine-mediated polyol process.^8^ Mohanty and colleagues presented an approach for one-pot, high-yield synthesis of hydrophilic nanoflowers of Pt as well as of other metals in the presence of an amino acid-based surfactant.^12^ Moreover, a sonoelectrodeposition method was developed by Heli and co-workers to produce Pt NFs hierarchically constructed from nanoparticles.^13^ We have recently reviewed the synthetic routes to obtain noble metal NPs (including Pt) with controlled composition, morphology, crystallinity and either a hydrophobic or hydrophilic surface.^14^

Hydrophilic Pt nanostructures are efficient catalysts for various reactions such as electrocatalytic oxidation of methanol,^15^ reduction of oxygen,^11^ and the Suzuki–Miyaura and Heck coupling reactions.^16^ Of much recent interest is the catalytic reduction of 4-nitrophenol (4-NP) to 4-aminophenol (4-AP), not only as a model reaction but also because nitrophenol is toxic in aqueous medium. One of the processes that have been proposed for nitrophenol degradation is the reduction of the nitro group to an amine, and spherical Pt nanoparticles, either hydrophilic^17^ or hydrophobic, supported onto a cellulose substrate^18^ have been reported to catalyse this reaction.

In catalytic reactions, most substrates and products present poor water solubility. Although this is one of the major limitations of water in organic chemistry, it can be turned into an advantage, not just because water is not toxic, safe, inexpensive and can accelerate organic reactions,^19^ but it also facilitates separation, recycling and reuse of water-soluble catalysts.^20^

We present here a one-pot method to prepare hydrophilic Pt nanoflowers, based on the solvothermal reduction of platinum acetylacetonate in diethylene glycol (DEG), in the presence of polyethylenimine (PEI). Since the reducing power of DEG depends on the reaction temperature, we first analyzed the effect of temperature on the size and shape of the obtained particles. We then analysed the importance of DEG in NF formation, through a comparison with *N*,*N*-dimethylformamide (DMF) as an alternative solvent and reducing agent. Anisole was also used for comparison, considering its role as a rather ‘inert’ solvent, with no reducing abilities. Apart from using conventional techniques such as X-ray diffraction and transmission electron microscopy, advanced electron tomography was also employed to obtain a 3D atomic scale reconstruction of the nanoparticles, so as to reveal their shape and crystallographic structure. We further analyzed the catalytic efficiency of the Pt nanoflowers in the hydrogenation of 4-nitrophenol (4-NP) to 4-aminophenol (4-AP) by sodium borohydride.^21^

## Experimental

### Materials

Branched PEI (Mw ∼ 25 000), ethanol, anhydrous anisole (99.7%), NaBH_4_ and 4-NP were provided by Sigma-Aldrich. Pt(acac)_2_ (98%) was obtained from Strem Chemicals Inc., NaOH was from Merck and DEG was from Fisher Sci. (analytical grade, 99.98%). All chemicals were used as received.

### Synthesis of Pt nanoflowers

30 mL of DEG containing 600 mM PEI (the concentration refers to monomer units) were introduced into a three-neck flask. After sonication and magnetic stirring at room temperature for 20 min, 0.1 mmol of Pt(acac)_2_ was added, forming a homogeneous solution with a yellowish colour. Then, the solution was rapidly heated to either 154 °C or 240 °C (heating rate ∼15 °C min^–1^). Within one hour at the selected temperature, the solution turned brown-black and it was kept at 154 °C or 240 °C for totally 24 h or 1 h, respectively. Finally, it was allowed to cool down to room temperature. The Pt NFs were precipitated twice by centrifugation with excess ethanol (8500 rpm, 30 min). Eventually, the nanoparticles were stored in 20 mL of Milli-Q water. The final Pt concentration was 3 mM in the case of the synthesis at 154 °C and 4 mM in the case of that at 240 °C, due to some slight difference in the reaction yield.

### Characterisation

Conventional TEM imaging was carried out using a JEOL JEM 1010 microscope operated at an acceleration voltage of 100 kV. The electron tomography series were acquired using a FEI Tecnai G2 electron microscope operated at 200 kV. A Fischione model 2020 single tilt tomography holder was used, and the series were acquired automatically with the use of Xplore3D software. All tilt series were acquired in HAADF-STEM mode with an angular range from –68° to +76° and a tilt increment of 2°. The alignment of the series was performed using the Inspect 3D software (FEI). The reconstruction of the series was performed by using the Simultaneous Iterative Reconstructive Technique (SIRT) as implemented in Inspect 3D. High resolution HAADF-STEM images were acquired using an aberration corrected cubed FEI Titan 50-80 electron microscope operated at 120 kV. The camera length was set to 115 mm in order to guarantee an incoherent imaging mode. Prior to using the HAADF-STEM images as inputs for the tomographic reconstruction, a constant background value was subtracted. The high resolution reconstruction was obtained by minimizing the L1 norm of the object simultaneously with the projection distance, as explained in ref. 24. The colloidal solutions were dried overnight under dynamic vacuum to prepare solid powder samples suitable for X-ray diffraction (XRD) measurements. XRD was measured using a Siemens D-5000 diffractometer with Cu Kα radiation (1.54059 Å). *Z*-potential values were acquired *via* electrophoretic mobility measurements by taking the average of five measurements at the stationary level, using a Zetasizer Nano S (Malvern Instruments) with a He–Ne laser operating at 633 nm and a detection angle of 173° (4 mW). ICP-OES elemental analyses were performed after digestion of the samples with *aqua regia*. FTIR spectra were recorded using a Nicolet 6700 FTIR spectrometer with a resolution of 4 cm^–1^ using previously dried powder of platinum nanoflowers in the form of KBr-based pellets.

### 4-Nitrophenol to 4-aminophenol reduction

In a 1 cm quartz cuvette, 1.34 mL of Milli-Q water was added, followed by the addition of 400 μL of an aqueous solution containing 0.1 mg of Pt NFs (21 nm). Then, 750 μL of a freshly prepared 0.1 M NaBH_4_ solution (9.1 mM in NaOH) was added, followed by 13 μL of 9.61 mM 4-nitrophenol aqueous solution. A diode-array UV-visible spectrophotometer (Agilent 8453) was employed to monitor the reaction by recording absorbance spectra every 30 s, at room temperature. The 4-aminophenol product was identified by monitoring the changes with time of the absorbance at 400 nm, where the maximum variations take place. The apparent observed rate constants *k*_app_ were calculated from the plots of absorbance *vs.* time, using the first-order rate equation:*A*_*t*_ = *A*_f_ + Δ*A* e^–*kt*^where *A*_*t*_ is the absorbance of the reaction mixture at time *t*, *A*_f_ is the final absorbance and *k* is the apparent observed first-order rate constant of the reaction.

### Recyclability test

When the catalytic reduction of 4-NP to 4-AP was deemed completed, 13 μL of 4-NP was added again to the reaction solution, and the kinetic studies were continued after mixing. This procedure was repeated several times.

## Results and discussion

**Fig. 1:**
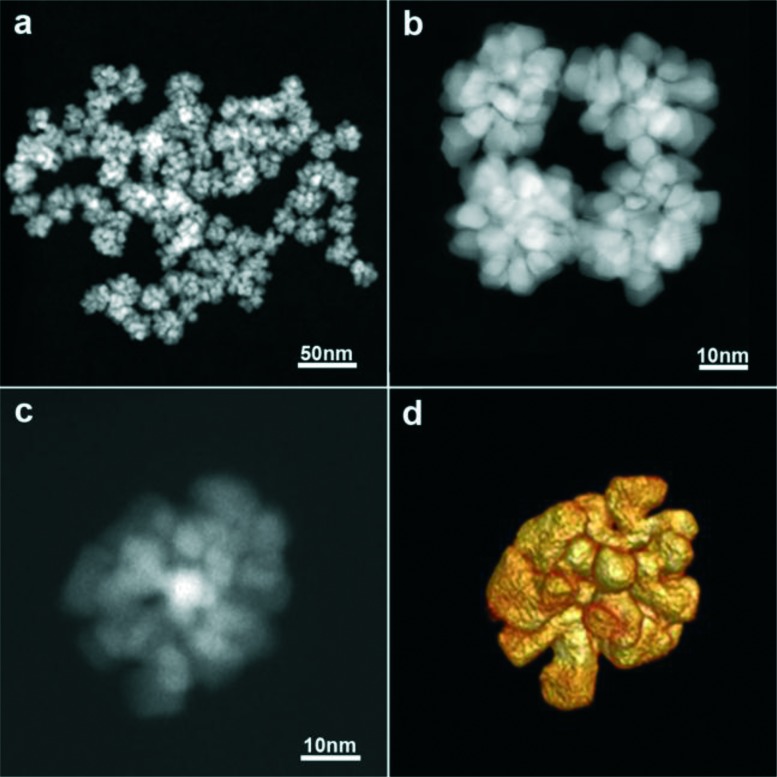
a–c) HAADF-STEM images of the Pt nanoflowers and d) a 3D representation of the reconstructed volume of the nanoflower shown in c.

Uniform platinum nanoflowers (Fig. 1 and S1†) were obtained by the reduction of Pt(acac)_2_ in a PEI-containing DEG solution at high temperature (see Experimental). DEG is expected to act as both a solvent and a reducing agent, whilst PEI is a capping agent providing high colloidal stability in water (*ζ* ∼ +40 mV), even for several months. The presence of PEI on the particle surface was further characterized by FTIR spectroscopy. As shown in Fig. S2,† the spectrum displayed peaks at 2925 and 2852 cm^–1^, assigned to the asymmetric and symmetric vibrations of the CH_2_ group, respectively, and at 1450 cm^–1^, attributed to the in-plane bending of the CH_2_ in PEI. Besides, the peaks corresponding to the bending vibration of the NH group (1635 cm^–1^) and the stretching vibration of the C–N groups (1160 cm^–1^) of PEI were clearly distinguished.^22^ In addition, the high boiling point of DEG (244–245 °C) allows us to use it within a wide temperature range, thus modifying its reducing capability; a higher reaction temperature allows sufficient rearrangement of atoms during growth, resulting in highly crystalline materials.^23^ The reaction was thus performed at 154 and 240 °C (Fig. 1 and S1†), showing that the particle morphology and uniformity were not affected, but particle size was slightly modified (21 nm *vs.* 34 nm, on average, for 154 °C and 240 °C, respectively), and the reaction kinetics were markedly different (24 h *vs.* 1 h, respectively).

**Fig. 2:**
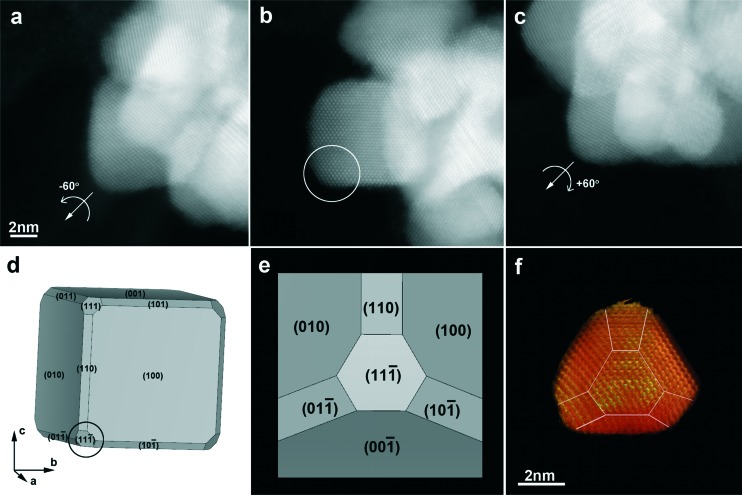
a–c) High resolution HAADF-STEM images of the branch used for the atomic scale reconstruction, oriented along three different <110> zone axes. The reconstructed part of the branch is the lower left corner, indicated by the white circle in b. d) A model of a truncated cube made using VESTA, which is the most dominant morphology in the branches. The cube is on the same orientation as the branch in Fig. 2b. e) The model along the [11–1] orientation, in order to be compared with the reconstructed volume, which is also oriented in the same direction. f) 3D representation of the reconstructed volume of the corner of the branch, where the indexing of the facets is also included. The facets are mainly {110} and {100}, and for the acquisition of the high resolution series, the branch was tilted around the corner of the cube, the [11–1] axis. In a, the crystal is tilted –60° with respect to b, while in c, it is tilted +60°.

Analysis by high angle annular dark-field scanning transmission electron microscopy (HAADF-STEM) showed that the NFs consist of branches and leaves with different crystallographic orientations. Since STEM images only provide 2D projections, electron tomography was used to obtain reliable 3D characterization of the morphology of the Pt NFs. As shown in Fig. 1c and d, the 3D reconstruction of a representative Pt NF shows that the branches are not thin leaves but rather exhibit a faceted morphology. XRD characterization additionally reveals that the NFs comprise high purity face-centred cubic (fcc) crystals (Fig. S3†). The XRD peaks matched well with Pt JCPDS card no.: 00-004-0802 and can be assigned to the crystal planes (111), (200), (220), and (311). Application of Scherrer's equation for the (111) peak at 39.8° yielded a crystalline monodomain size of around 7 nm, which is in agreement with the values obtained by TEM for the size of a single “petal”. Taking into account that TEM analysis gives an average particle size of 21 or 34 nm (for 154 and 240 °C, respectively), we expect that the NFs comprise multiple crystallographic domains, which was confirmed by high-resolution STEM. Fig. S4† clearly shows that the petals display different crystallographic orientations with twin boundaries between them. Aiming to obtain a better understanding of the morphology of the individual petals comprising the whole NF, a 3D reconstruction at the atomic scale was obtained (Fig. 2 and S5†). Three high resolution images from a tip of a petal were acquired along different <110> zone axes (Fig. 2a–c) and then used as input for a compressive sensing-based reconstruction algorithm.^24^ As can be observed in Fig. 2, the morphology of the investigated petal corresponds to a truncated cube. A model of such a cube, generated using VESTA (a 3D visualization program for electronic and structural analysis),^25^ is presented in Fig. 2d, which is the most dominant morphology in the branches. This comprehensive morphological and crystallographic investigation clearly demonstrated the ‘faceted’ configuration of the petals, which are thus the ‘constituting units’ for the NFs.

Our results are different from those presented by Yin *et al.*,^8^ where the use of iodine ions resulted in the formation of monomorphic single-crystalline Pt nanoflowers. In their case, the continuous fringes from the core to the petals suggest that the latter grow epitaxially, and all the Pt atoms are arranged in one periodicity, providing a uniform orientation for all the branches or petals.^8,12^ The polycrystalline nature of our nanoflowers indicates a distinct formation mechanism. It is likely that the individual nanocrystals coalesce at the early stages due to competition between the remaining Pt precursors and PEI (in constant attachment/detachment induced by the high temperature).^7^ Such a ‘coalescence’ mechanism may be due to a slow reduction rate, resulting in a large amount of monomers that crystallize into a large number of nuclei, which are near each other in an environment rich in Pt monomers. This situation facilitates a ‘coalescence’ growth mode, leading to progressive condensation of the nanocrystals into the nanoflower structures.^7^ The presence of PEI helps controlling the extent of such coalescence, hindering the formation of large platinum aggregates.

The importance of using DEG as a solvent was confirmed by carrying out the reaction in either DMF or anisole, keeping all the other conditions unchanged. Nanoparticles with a dendrite shape were obtained with DMF (in agreement with ref. 26, Fig. S6a†), whereas oligopods were the main morphology when anisole was used (Fig. S6b†). The differences in oxidation potential and/or coordination behaviour in various solvents have been reported to result in varying sizes and shapes.^27–30^

**Fig. 3:**
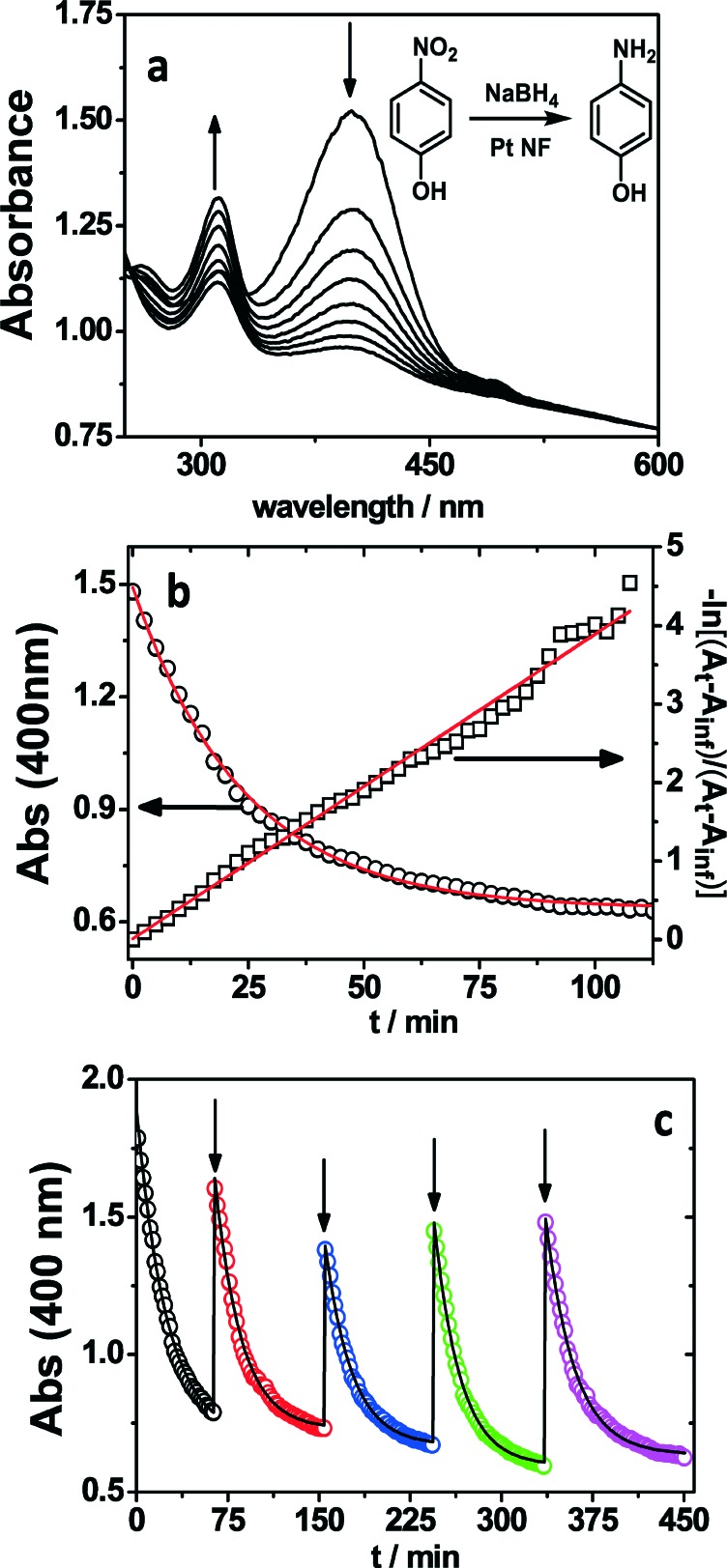
a) Spectral evolution of a mixture of 4-NP and Pt nanoflowers upon borohydride addition. [4-NP] = 0.05 mM, 0.1 mg of Pt NF and [NaBH_4_] = 0.03 M, *T* = 25 °C. b) Kinetic trace of the absorbance at 400 nm during the reduction of 4-NP, and linearized data for first-order analysis corresponding to Fig. 3a. c) Absorbance kinetic traces at 400 nm, registered during the sequential reduction of 4-NP. The arrows indicate the times at which 4-NP was added to obtain [4-NP] = 0.05 mM. The line represents the best fit to a first-order rate equation.

Finally, the catalytic performance of 21 nm platinum nanoflowers was tested for the conversion of 4-nitrophenol (4-NP) to 4-aminophenol (4-AP) by sodium borohydride (see the inset in Fig. 3a). 4-Aminophenol is an important intermediate in the preparation of drugs, lubricants, and dyes.^3,31^ Therefore, it is desirable to develop efficient and reusable catalytic systems for the preparation of 4-AP. This reaction can be easily monitored by UV-visible spectroscopy, since nitrophenolate ions and 4-AP display absorption bands centred at 400 nm and 300 nm, respectively (Fig. 3a). Although sodium borohydride is regarded as a strong reducing agent, it can be considered inert for 4-nitrophenol reduction in the absence of a catalyst. The presence of the Pt NFs in the reaction medium leads to a gradual decrease of the band for nitrophenolate ions, along with a gradual increase in intensity of the 4-AP band, which indicates the catalytic conversion of 4-NP to 4-AP (Fig. 3a). It should be pointed out that a large excess of NaBH_4_ with respect to 4-NP allows us to treat the kinetics of the catalytic process as a pseudo-first order reaction. The apparent rate constant (*k*_app_) was assumed to be proportional to the surface *S* of the metal and also to the sodium borohydride concentration, therefore, the kinetic constant (*k*_app_) can be defined as1
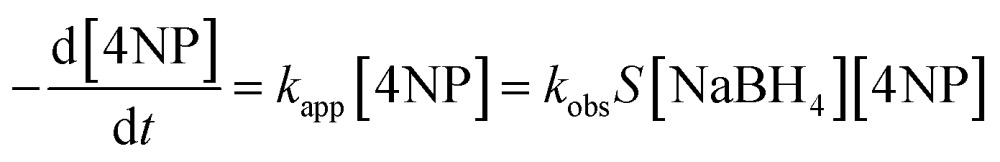
where *k*_obs_ is the observed pseudo-first order rate constant, *S* is the metal catalyst surface, [4-NP] is the concentration of 4-NP at a given time *t* and [NaBH_4_] is the sodium borohydride concentration (assumed to be constant).

Fig. 3b shows the reaction kinetics through a plot of the absorbance at 400 nm *vs.* reaction time, as well as a fit to a first-order rate equation (see the experimental section), which yields an observed rate constant of 7.0 × 10^–4^ s^–1^. Additionally, the linearized data for first-order analysis also highlight the high-quality first-order nature of the reaction. In order to compare our results with previous reports, the normalized rate constant (*k*_nor_) was determined by

2 *k*_nor_ = *k*_app_/(*m*[NaBH_4_])

normalizing *k*_app_ with respect to the total amount of the catalyst (*m*) and the borohydride concentration ([NaBH_4_]). The metallic Pt content was determined by ICP-OES spectroscopy to be 0.1 mg. Thus, the *k*_nor_ value obtained for the Pt NFs (21 nm) was *ca.* 233.3 g^–1^ s^–1^ M^–1^. This value is greater than the *k*_nor_ estimated for other nanoparticle-based catalysts, such as Pt black (69 g^–1^ s^–1^ M^–1^),^32^ Au@citrate (27.6 g^–1^ s^–1^ M^–1^)^32^ or Ag (68.9 g^–1^ s^–1^ M^–1^)^33^ and Au (77.5 g^–1^ s^–1^ M^–1^)^34^ dendrites (see Table S1†), indicating their superior performance.

To check the reproducibility/reusability of the Pt NFs as catalysts, the nitro to amine conversion was repeated several times, using the same colloidal dispersion, through the sequential addition of 4-NP to an aqueous solution containing excess borohydride and a constant concentration of the platinum nanoflowers. Fig 3c shows the kinetic trace at 400 nm as well as the fit to a pseudo first-order reaction for each 4-NP addition. It is obvious from this plot that the first-order kinetic behaviour was perfectly reproducible, as predicted by eqn (1). The catalyst reusability with a yield of 100% 4-AP after the fifth cycle indicated no loss in catalytic activity. Moreover, the consistency of the calculated apparent rate constant, *k*_app_, alongside the different additions/cycles (5 in total) of 4-NP (7.0 × 10^–4^ s^–1^, 7.8 × 10^–4^ s^–1^, 7.7 × 10^–4^ s^–1^, 7.8 × 10^–4^ s^–1^, and 7.0 × 10^–4^ s^–1^) is a clear evidence for the remarkable stability of the Pt NFs in the reaction medium, as well as the reproducibility of their catalytic activity. Typically, the deactivation of catalytic efficiency during recycling, when dealing with colloidal catalysts, has been ascribed to the agglomeration of the metal nanoparticles leading to a decrease in the active surface area.^34^ In our case, the high catalytic reusability can be ascribed to the high stability provided by the positively charged polymer PEI, even at high ionic strength. The Pt nanoparticles were observed by TEM after the catalytic cycles and, as shown in Fig. S7,† the reduction of 4-NP did not affect the nanoflower morphology.

## Conclusions

To conclude, uniform platinum nanoflowers were synthesized *via* a one-pot protocol based on the solvothermal reduction of Pt(acac)_2_ in DEG, employing PEI as a capping agent. The presence of PEI on the particle surface provides them high stability in water. Complete structural characterization *via* HAADF-STEM and 3D reconstruction by electron tomography showed that the NFs consisted of multiple fcc branches (around 7 nm) with a truncated cubic morphology and different crystallographic orientations. Analysis of the catalytic reduction of 4-nitrophenol to 4-aminophenol with sodium borohydride showed superior catalytic activity of the Pt NFs compared to other metal nanoparticles, as well as good reusability.
