# Supplementary material for: Hydrophilic Pt nanoflowers: synthesis, crystallographic analysis and catalytic performance
Source: CrystEngComm. 2016 Apr 12;18(19):3422–7. doi: 10.1039/c6ce00039h (PMC5361136; doi:10.1039/c6ce00039h)
Supplement: Supplementary file 1 [file CE-018-C6CE00039H-s001.pdf]

Electronic Supplementary Material (ESI) for CrystEngComm.
This journal is © The Royal Society of Chemistry 2016

Electronic Supplementary Material

Hydrophilic Pt nanoflowers: synthesis, crystallographic analysis and catalytic performance

Stefanos Mourdikoudis,[a] Thomas Altantzis,[b] Luis M. Liz-Marzán,[a,c,d] Sara Bals,[b] Isabel Pastoriza-Santos[a] and Jorge Pérez-Juste[a]

[a] *Departamento de Química Física, Universidade de Vigo, 36310 Vigo, Spain.*

[b] *EMAT, University of Antwerp, Groenenborgerlaan 171, B-2020 Antwerp, Belgium*

[c] *BioNanoPlasmonics Laboratory, CIC biomaGUNE, Paseo de Miramón 182, 20009 San Sebastián, Spain*

[d] *Ikerbasque, Basque Foundation for Science, 48013 Bilbao, Spain*

Corresponding authors' e-mail : juste@uvigo.es, pastoriza@uvigo.es

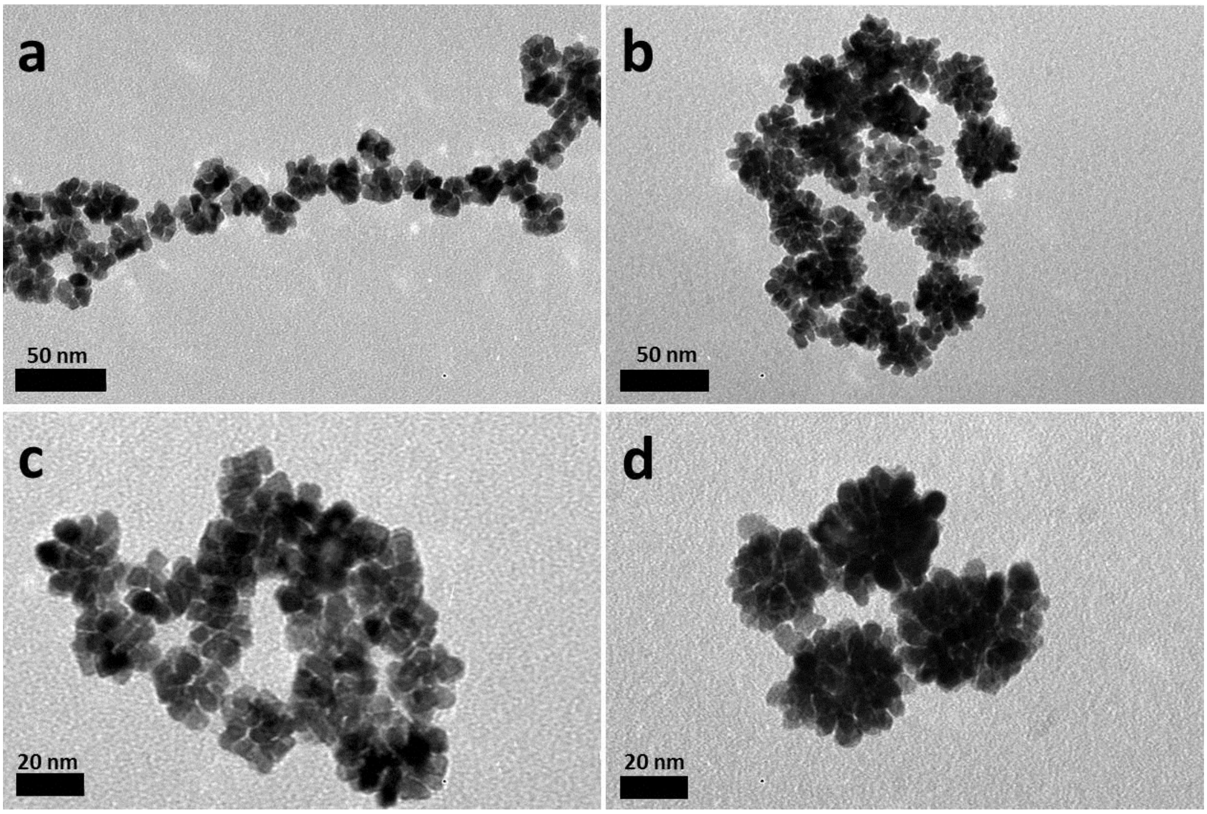

Fig. S1 TEM images of ~ 21 nm (a, c) and 34 nm (b, d) Pt nanoflowers obtained via reduction of $Pt(acac)_2$ in DEG containing PEI. The reaction temperature was 154 ºC (a, c) and 240 ºC (b, d).

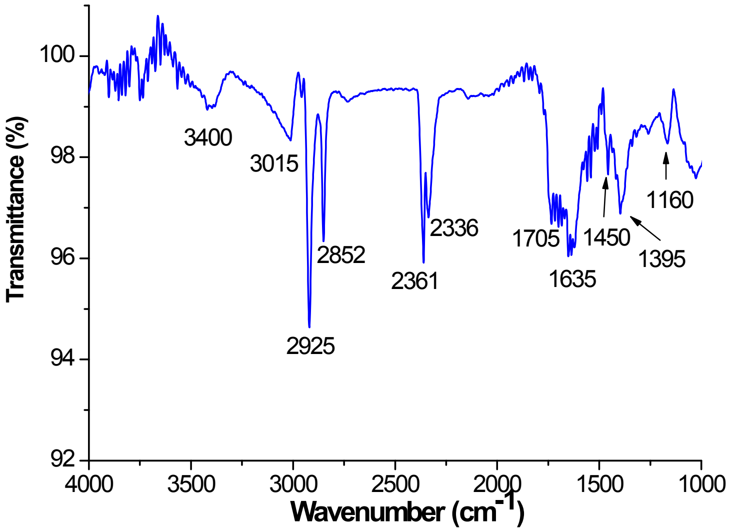

Fig. S2 FTIR spectrum of platinum nanoflowers. The peaks at 2925 and 2852 cm^{-1} are assigned to the asymmetric and symmetric vibration of the CH_2 group in PEI, while the peak at 1450 cm^{-1} is attributed to in-plane bending of CH_2. The peaks corresponding to the bending vibration of the NH group and the stretching vibration of the C-N groups of PEI are located at 1635 and 1160 cm^{-1}, respectively.[1] The two peaks around 2350 cm^{-1} are probably present because of the vO=C=O from the CO_2 in the ambient air. Regarding the broad peak at 3400 cm^{-1}, it is due to the O-H stretching in the H_2O molecule, which is present despite the drying of the sample to record the FTIR spectra.

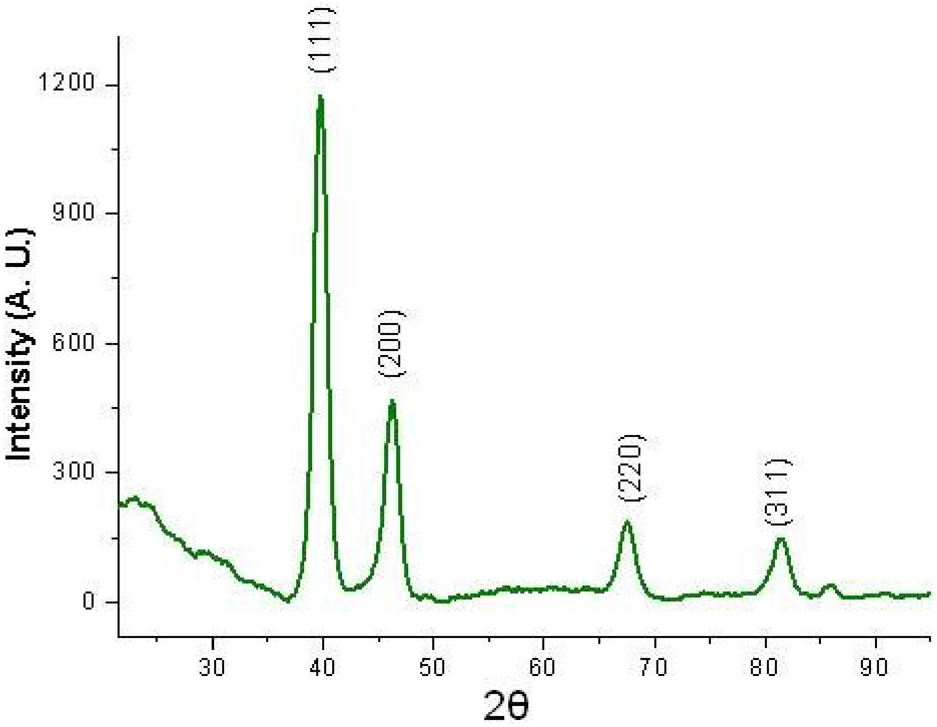

Fig. S3 Typical XRD measurement of Pt nanoflowers. The fcc-Pt peaks are evident. The peaks at 39.8, 46.3, 67.5 and 81.5^{o} can be easily assigned to the (111), (200), (220) and (311) lattice planes of fcc-Pt (JCPDS card No. 04-0850), showing a high degree of metallic character and crystallinity for our nanomaterials. The (111) peak is much stronger than the (200) peak, which is an expected observation for randomly-oriented 3D assembly of Pt nanomaterials.

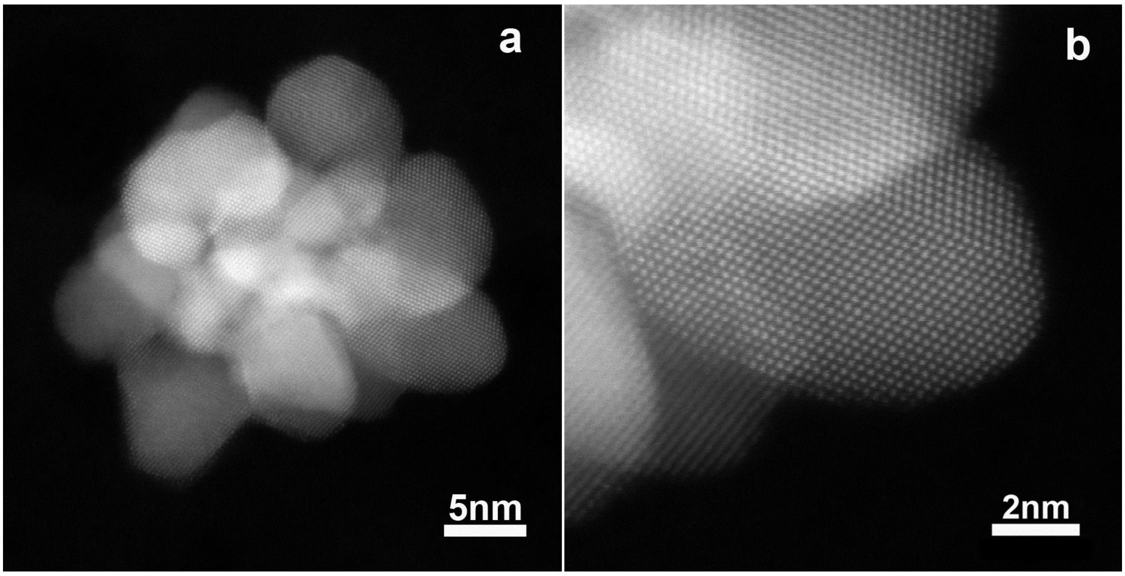

Fig. S4 a) and b) High resolution HAADF-STEM images confirming that the flowers consist of branches with different crystallographic orientations. The existence of twin boundaries is prevalent.

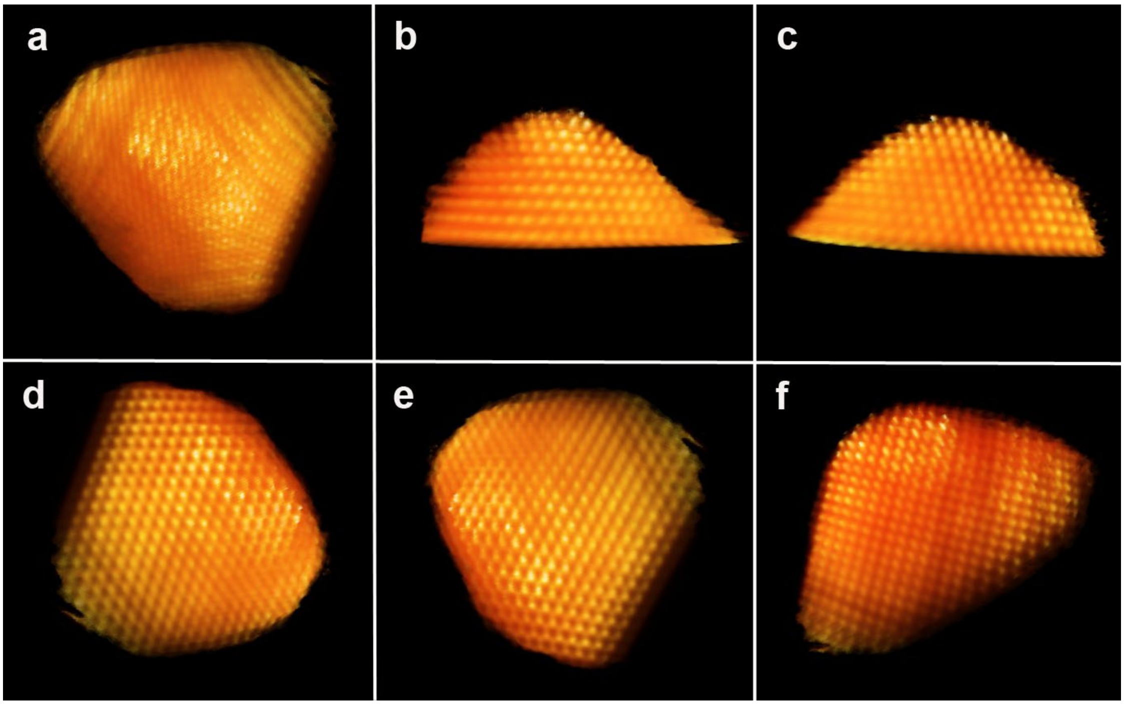

Fig. S5 3D representation of the reconstructed volume which is depicted in Fig. 2f of the main manuscript, along different views.

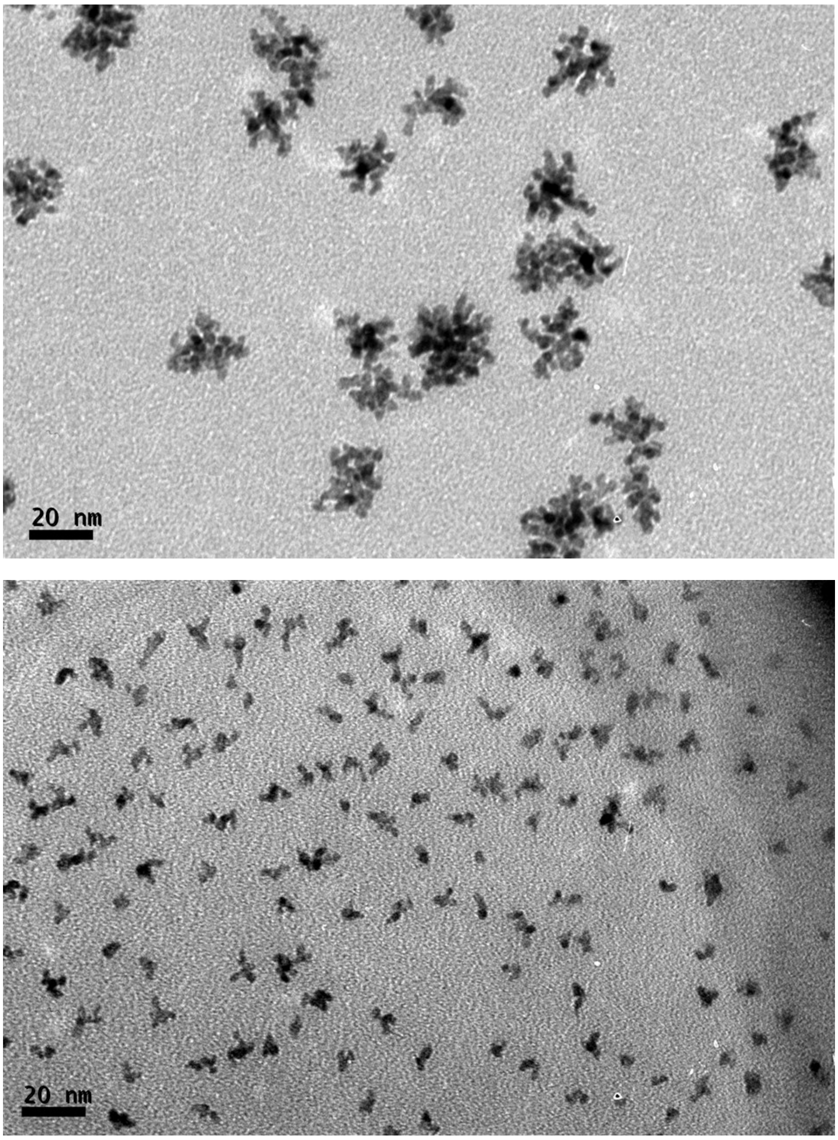

Fig. S6 TEM images of platinum nanostructures obtained in DMF (a) and anisole (b) instead of DEG. The rest of experimental conditions (concentration of $Pt(acac)_2$ and PEI, temperature (154 ^oC) were maintained unchanged. These nanostructures form stable colloidal solutions in aqueous medium for several months, as in the case of the Pt nanoflowers.

Table S1 Summary of catalytic performances of different nanomaterials employed for the reduction of 4-NP by $NaBH_4$.

Catalyst	k_{app} (s^{-1})	k_{nor} (g s^{-1} M^{-1})	Ref.
Pt Nanoflowers	7.0×10^{-4}	233.3	This work
Pt black	7.0×10^{-4}	69.0	2
Au@citrate	3.0×10^{-4}	27.6	2
Ag dendrites	2.51×10^{-3}	68.9	3
Au dendrites	2.82×10^{-3}	77.5	3

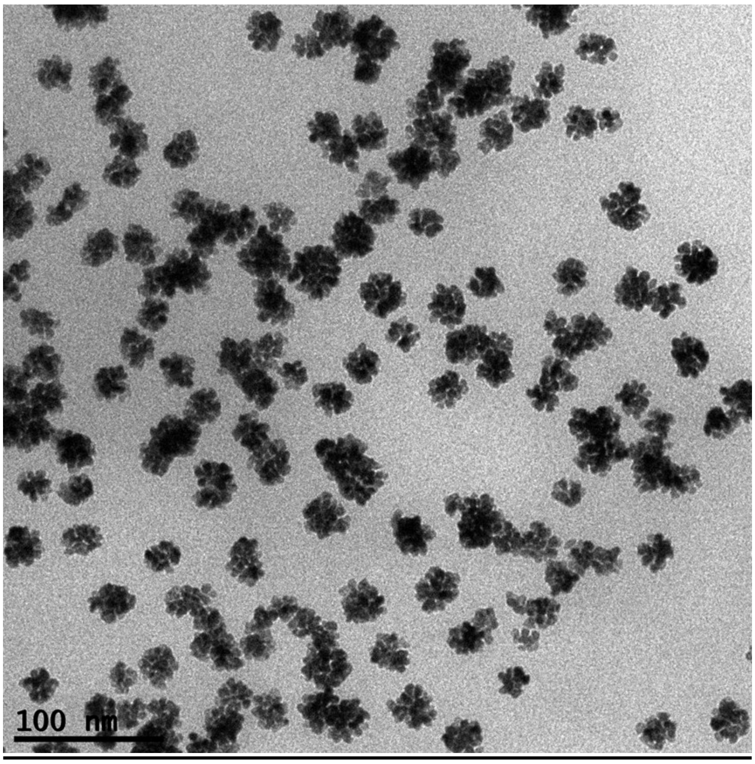

Fig. S7 TEM images of Pt nanoflowers after the catalytic reduction of 4-NP by $NaBH_4$.

REFERENCES

[1] T. Yang, A. Hussain, S. Bai, I. A. Khalil, H. Harashima and F. Ahsan, *J. Control. Release.,* **2006**, *115*, 289.

[2] .-J. Lv, A.-J. Wun, X. Ma, R.-Y. Xiang, J.-R. Chen and J.-J. Feng, *J. Mater. Chem. A* 2015, **3**, 290.

[3] W. Ye, Y. Chen, F. Zhou, C. Wang and Y. Li, *J. Mater. Chem.* 2012, **22**, 18327
